# Supplementary material for: Cryo-EM structure of a type IV secretion system
Source: Nature. 2022 Jun 22;607(7917):191–6. doi: 10.1038/s41586-022-04859-y (PMC9259494; doi:10.1038/s41586-022-04859-y)
Supplement: Supplementary file 1 — Uncropped gel and western blot images. [file 41586_2022_4859_MOESM1_ESM.pdf]

nature portfolio

https://doi.org/10.1038/s41586-022-04859-y

Supplementary information

Cryo-EM structure of a type IV secretion system

In the format provided by the authors and unedited

SI Fig. 1

Cryo-EM structure of a type IV secretion system

Kévin Macé, Abhinav K. Vadakkepat, Adam Redzej, Natalya Lukoyanova, Clasien Oomen, Nathalie Braun, Marta Ukleja, Fang Lu, Tiago R.D. Costa, Elena V. Orlova, David Baker, Qian Cong, and Gabriel Waksman

SI Fig. 1: uncropped gel and western blot images. See legends and contents of corresponding ED Figure panels for lane and band identifiers. Areas shown in corresponding ED figures are indicated within a dashed line box. MW (kDa) are indicated. a, Raw data gel image for Fig. 1c. b, Raw data gel image for ED Fig. 1d. c, Raw data gel and western blot images for ED Fig. 10c. Upper panel: raw data gel image for ED Fig. 10c top panel; middle panel: raw data western blot image for ED Fig. 10c middle panel; lower: raw data western blot image for ED Fig. 10c lower panel. d, Raw data western blot images for ED Fig. 10h. Upper panel: raw data western blot image for ED Fig. 10h left panel; lower panel: raw data western blot image for ED Fig. 10h right panel. Controls were run on the same gel as loading controls.

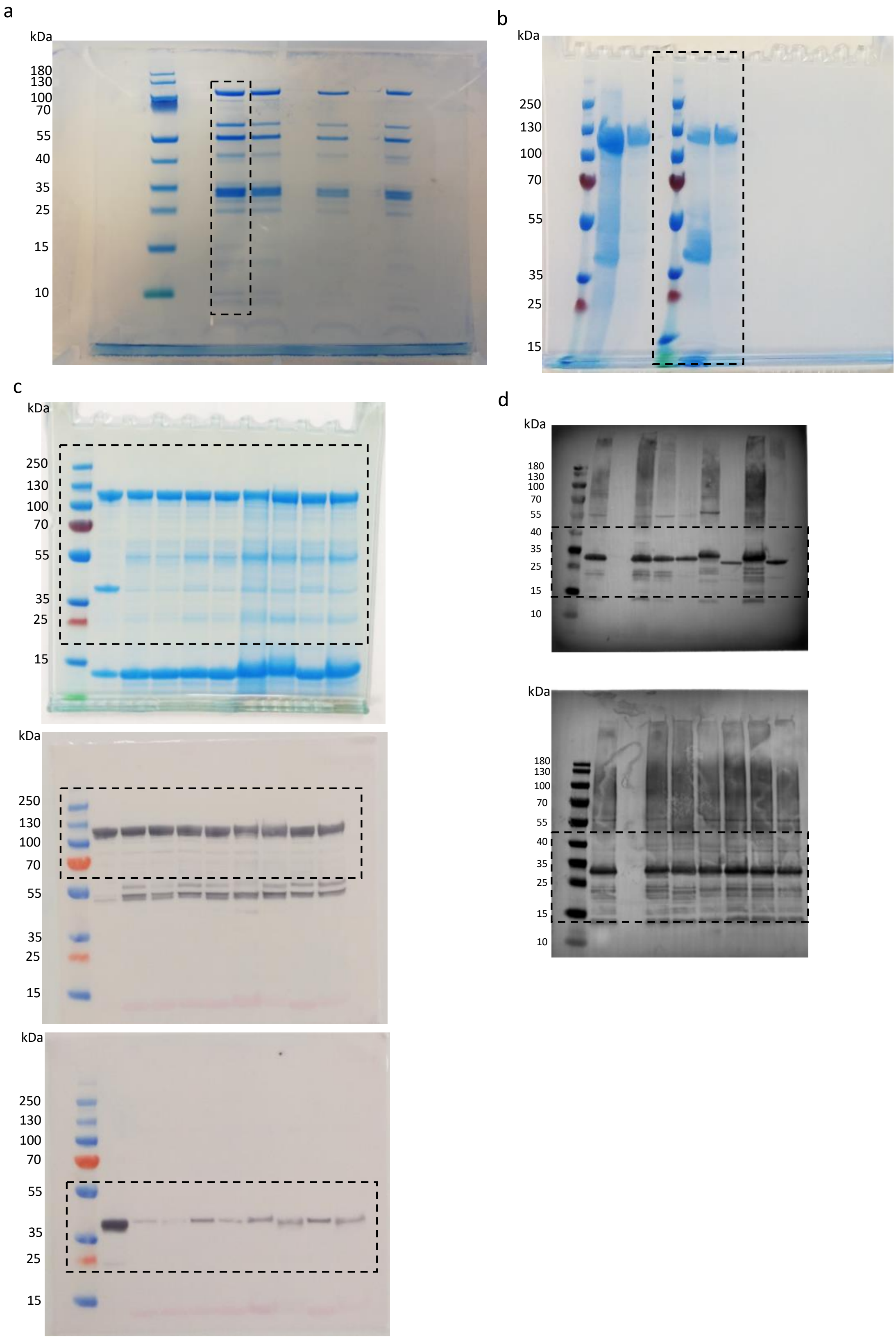
a
b
c
d
kDa
180
130
100
70
55
40
35
25
15
10
250
