# Supplementary material for: Cryo-EM structure of a type IV secretion system
Source: Nature. 2022 Jun 22;607(7917):191–6. doi: 10.1038/s41586-022-04859-y (PMC9259494; doi:10.1038/s41586-022-04859-y)
Supplement: Supplementary file 2 — Reporting Summary [file 41586_2022_4859_MOESM2_ESM.pdf]

nature research

Corresponding author(s): Gabriel Waksman, Kévin Macé, Qian Cong

Last updated by author(s): May 7, 2021

Reporting Summary

Nature Research wishes to improve the reproducibility of the work that we publish. This form provides structure for consistency and transparency in reporting. For further information on Nature Research policies, see our Editorial Policies and the Editorial Policy Checklist.

Statistics

For all statistical analyses, confirm that the following items are present in the figure legend, table legend, main text, or Methods section.

n/a	Confirmed	
☐	☒	The exact sample size (*n*) for each experimental group/condition, given as a discrete number and unit of measurement
☐	☒	A statement on whether measurements were taken from distinct samples or whether the same sample was measured repeatedly
☐	☒	The statistical test(s) used AND whether they are one- or two-sided<br>*Only common tests should be described solely by name; describe more complex techniques in the Methods section.*
☒	☐	A description of all covariates tested
☒	☐	A description of any assumptions or corrections, such as tests of normality and adjustment for multiple comparisons
☐	☒	A full description of the statistical parameters including central tendency (e.g. means) or other basic estimates (e.g. regression coefficient) AND variation (e.g. standard deviation) or associated estimates of uncertainty (e.g. confidence intervals)
☐	☒	For null hypothesis testing, the test statistic (e.g. *F*, *t*, *r*) with confidence intervals, effect sizes, degrees of freedom and *P* value noted<br>*Give P values as exact values whenever suitable.*
☒	☐	For Bayesian analysis, information on the choice of priors and Markov chain Monte Carlo settings
☒	☐	For hierarchical and complex designs, identification of the appropriate level for tests and full reporting of outcomes
☒	☐	Estimates of effect sizes (e.g. Cohen's *d*, Pearson's *r*), indicating how they were calculated

Our web collection on statistics for biologists contains articles on many of the points above.

Software and code

Policy information about availability of computer code

Data collection	EPU version 2.7 software (Thermo Fisher, USA), HMMER4
Data analysis	RELION 3.1, CRYOSPARC v2.15, MOTIONCOR2 , CTFFIND v4.1 , GAUTOMATCH v0.563, PHENIX v1.18, PHENIX v1.18.2, MOLPROBITY v4.4, MOLPROBITY v4.5.1 , COOT v0.9.3, CHIMERA v1.13.1, CHIMERA v1.4, CHIMERAX v1.1, PYMOL v2.3.2, HDOCK webserver, PATCHDOCK webserver, YASARA webserver, HMMER hmmbuild webserver, ROSETTA webserver, ROSETTACM webserver, TrRosetta.v1, ROBETTA webserver, PSIPRED webserver, PHYRE webserver, PISA webserver, TMpred webserver, DALI webserver, ALPHAFOLD1, FIJI-1.53, IMAGIC-5.

For manuscripts utilizing custom algorithms or software that are central to the research but not yet described in published literature, software must be made available to editors and reviewers. We strongly encourage code deposition in a community repository (e.g. GitHub). See the Nature Research guidelines for submitting code & software for further information.

Data

Policy information about availability of data

All manuscripts must include a data availability statement. This statement should provide the following information, where applicable:

- Accession codes, unique identifiers, or web links for publicly available datasets
- A list of figures that have associated raw data
- A description of any restrictions on data availability

EM maps and atomic models were deposited to the EMDB and PDB data bases. Accession codes can be found in ED Table 1 of the manuscript. PDB codes for the various structures reported in this manuscript are 7O3J, 7O3T, 7O3V, 7O41, 7O42, 7O43, 7OIU, 7Q1V and the EMDB deposition numbers for the EM maps are EMD-12707, EMD-12708, EMD-12709, EMD-12715, EMD-12716, EMD-12717, EMD-13765, EMD-13766, EMD-13767, EMD-13768, EMD-12933. All constructs (wild-type and mutants) used in this study can be obtained on request to GW.

Field-specific reporting

Please select the one below that is the best fit for your research. If you are not sure, read the appropriate sections before making your selection.

☒ Life sciences ☐ Behavioural & social sciences ☐ Ecological, evolutionary & environmental sciences

For a reference copy of the document with all sections, see nature.com/documents/nr-reporting-summary-flat.pdf

Life sciences study design

All studies must disclose on these points even when the disclosure is negative.

Sample size	104,711 micrographs were collected.
Data exclusions	No data were excluded from the analysis
Replication	All conjugation measurements were performed in triplicates
Randomization	Not relevant to this study
Blinding	Not relevant to this study

Reporting for specific materials, systems and methods

We require information from authors about some types of materials, experimental systems and methods used in many studies. Here, indicate whether each material, system or method listed is relevant to your study. If you are not sure if a list item applies to your research, read the appropriate section before selecting a response.

Materials & experimental systems

n/a	Involved in the study
☐	☒ Antibodies
☒	☐ Eukaryotic cell lines
☒	☐ Palaeontology and archaeology
☒	☐ Animals and other organisms
☒	☐ Human research participants
☒	☐ Clinical data
☒	☐ Dual use research of concern

Methods

n/a	Involved in the study
☒	☐ ChIP-seq
☒	☐ Flow cytometry
☒	☐ MRI-based neuroimaging

Antibodies

Antibodies used	Anti-His antibodies, Anti-Strep antibodies, Anti-FLAG antibodies
Validation	Anti-His antibodies were from SIGMA, Anti-Strep antibodies were from MERCK, anti-FLAG and anti-rabbit antibodies were from ABCAM. These commercially available antibodies are validated by the companies.
