# Supplementary material for: Cryo-EM structure of a type IV secretion system
Source: Nature. 2022 Jun 22;607(7917):191–6. doi: 10.1038/s41586-022-04859-y (PMC9259494; doi:10.1038/s41586-022-04859-y)
Supplement: Supplementary file 3 — This file contains Supplementary Tables 1-3. [file 41586_2022_4859_MOESM3_ESM.pdf]

SI Table 1-3

Cryo-EM structure of a type IV secretion system

Kévin Macé, Abhinav K. Vadakkepat, Adam Redzej, Natalya Lukoyanova, Clasien Oomen, Nathalie Braun, Marta Ukleja, Fang Lu, Tiago R.D. Costa, Elena V. Orlova, David Baker, Qian Cong, and Gabriel Waksman

SI Table 1a. Cryo-EM data collection and processing statistics and supplementary information. The supplementary information part of this table provides information on how each map was used, the part(s) that were built in each map when relevant, the method that was used to build the model from each map when relevant, and the PDB entry code of the corresponding model.

Complex/Subcomplex	T4SS										TrwK/VirB4unbound	
Name of the maps generated in this study	*Ab initio* model for OMCC C1	OMCC C1 3.28 Å	O-layer C14 2.58 Å	I-layer C16 3.08 Å	*Ab initio* model for IMC-Arches-Stalk	IMC-Arches-Stalk C1 6.18 Å	IMC protomer C1 3.75 Å	Stalk C1 3.71 Å	Stalk C5 3.28 Å	IMC-Arches C6 8.33 Å	Trimer of dimers C1 4.14 Å	Dimer C1 3.49 Å
EMDB entry	EMD-13765	EMD-13765	EMD-12707	EMD-12708	EMD-13766	EMD-13767	EMD-12933	EMD-12709	EMD-13768	EMD-12715	EMD-12716	EMD-12717
**Data collection and processing**												
Magnification	130,000										165,000	
Voltage (kV)	300										300	
Electron exposure (e⁻/Å²)	54										49	
Defocus range (μm)	-1.5 to -3.5										-1.2 to 2.8	
Pixel size (Å)	1.067	1.067	1.067	1.067	2.134	1.067	1.067	1.067	1.067	2.134	1.048	1.048
Symmetry imposed	C1	C1	C14	C16	C1	C1	C1	C1	C5	C6	C1	C1
Initial particle images (no.)	1,729,311	1,729,311	1,729,311	1,729,311	443,336	1,729,311	1,729,311	1,729,311	1,729,311	443,336	1,622,003	1,622,003
Final particle images (no.)	100,000	1,729,311	709,769	950,171	28,902	566,815	566,815	566,815	566,815	15,239	209,217	209,217
Map resolution (Å)	3.52	3.28	2.58	3.08	8.42	6.18	3.75	3.71	3.28	8.33	4.1	3.5
FSC threshold	0.143	0.143	0.143	0.143	0.143	0.143	0.143	0.143	0.143	0.143	0.143	0.143
Map resolution range (Å)	3.0-5.0	2.9-4.0	2.4-3.0	2.9-3.2	7.0-15	3.0-7.5	3.1-6.5	3.0-7.0	2.8-4.5	7.0-15	3.5-9.0	3.0-7.2
Map sharpening B factor (Å²)	-114.4	-168.8	-41.2	-80.4	-565.1	-468.7	119.7	-81.7	-87.03	-844.2	-126.1	-137

Additional information

Map was used to	As initial model for OMCC C1 3D refinement. Determine the O-layer symmetry	Establish the symmetry of O- and I-layers	Build the O-layer	Build the I-layer	As initial model for 3D classification and refinement	1- Establish symmetry of IMC, Stalk and Arches | 2- Initial reference for final reconstruction of the IMC protomer and the Stalk | 3- Fit TrwG/VirB8peri domains	Build the IMC protomer	Build the Stalk including membrane helices	Improve the periplasmic part of the Stalk	1- Determine the NCS operators to generate the IMC-Arches hexameric model | 2- Position the OMCC C1 map to generate the entire T4SS composite model	Build the trimer of dimer form of TrwK/VirB4unbound	Build the first structure of TrwK/VirB4
Part(s) built in the map	N/A	N/A	14xTrwH/ VirB7 14xTrwF/ VirB9CTD 14xTrwE/ VirB10CTD	16xTrwF/ VirB9NTD 16xTrwE/ VirB10NTD	N/A	3 x TrwG/ VirB8peri	1xTrwM/ VirB3 2xTrwK/ VirB4 3xTrwG/ VirB8tails	5xTrwJ/VirB5 5xTrwI/VirB6 with trans membrane helices	5xTrwJ/ VirB5 and periplasmic part of 5xTrwI/ VirB6	N/A	Build the trimer of dimers	TrwK/VirB4unbound dimer
Modelling method	N/A	Rigid body	Building and refinement	Building and refinement	N/A	Rigid body	Building and refinement	Building and refinement	Building and refinement	N/A	Rigid body	Building and refinement
PDB deposited	N/A	7O3J & 7O3T	7O3J	7O3T	N/A	7Q1V	7OIU	7O3V	7O3V	7O41	7O42	7O43

SI Table 1b. Model building and validation statistics.

Complex/Subcomplex	T4SS						TrwK/VirB4$_{unbound}$	
Structures generated in this study	O-Layer	I-Layer	Stalk	IMC protomer	Arches protomer	IMC-Arches hexameric model	TrwK/VirB4$_{unbound}$ trimer of dimers	TrwK/VirB4$_{unbound}$ dimer
PDB entry	7O3J	7O3T	7O3V	7OIU	7Q1V	7O41	7O42	7O43
**Refinement**[1]								
EMDB corresponding	EMD-12707	EMD-12708	EMD-12709	EMD-12715	EMD-13767	---	EMD-12716	EMD-12717
Initial model used (PDB code)	3ZBI	6GYB	*de novo*	*de novo*	2BHM	7O41 & 7Q1V	*de novo*	*de novo*
Model resolution (Å)	2.8	3.2	4	4	2.9	---	4.4	4.1
FSC threshold	0.5	0.5	0.5	0.5	0.5	---	0.5	0.5
CC Model vs. Data (mask)	0.82	0.83	0.74	0.77	0.62	---	0.79	0.71
**Model composition**								
Nonhydrogen atoms	38528	17456	15742	12744	1647	86328	43120	12.551
Protein residues	5068	2144	2153	1683	411	12564	5617	1630
Ligands	---	---	---	---	---	---	---	---
**B factors (Å^2)**								
Protein	70.53 (123 – 46)	45.8 (91 – 22)	43.42 (74 – 12)	90.45 (152 - 50)	0.99 (0.57 – 4)	80.23(0.57-152)	240.60 (611 – 76)	92.09 (194 – 42)
Ligand	---	---	---	---	---	---	---	---
**R.m.s. deviations**								
Bond lengths (Å)	0.008	0.011	0.004	0.004	0.008	0.006	0.006	0.005
Bond angles (°)	0.654	0.735	0.701	0.666	2.065	1.152	1.044	1.01
**Validation**[1]								
MolProbity score	1.37	1.6	1.73	1.85	0.96	1.8	2.05	2.01
Clashscore	5.76	10.41	11.15	10.07	1.92	10.02	9.69	6.29
Rotamer outliers (%)	0	0	0	0	0	0	0.78	0.32
**Ramachandran plot**								
Favored (%)	97.74	97.69	97.03	95.32	98.52	95.97	90.44	84.69
Allowed (%)	2.26	2.31	2.97	4.68	1.48	4.03	9.45	15.13
Disallowed (%)	0	0	0	0	0	0	0.11	0.18

SI Table 2. Interface area between 2 subunits. “molX” refer to the chain identifier given in the deposited PDB.

Interface between		Area (Å^2)
Name Molecule X	Name Molecule Y	
TrwK/VirB4$_{central}$	TrwK/VirB4$_{central}$	833
TrwK/VirB4$_{central}$	TrwK/VirB4$_{outside}$	1734
TrwK/VirB4$_{central}$	TrwM/VirB3	2805
TrwK/VirB4$_{central}$ adjacent	TrwM/VirB3	104
TrwK/VirB4$_{outside}$	TrwG/VirB8$_{tails}$ molA	732
TrwK/VirB4$_{outside}$	TrwG/VirB8$_{tails}$ molB	1236
TrwG/VirB8$_{tails}$ molA	TrwG/VirB8$_{tails}$ molB	803
TrwG/VirB8$_{tails}$ molB	TrwG/VirB8$_{tails}$ molC	506
TrwG/VirB8$_{tails}$ molA	TrwG/VirB8$_{tails}$ molC	310
TrwI/VirB6 molA	TrwI/VirB6 molE	1357
TrwJ/VirB5 molE	TrwJ/VirB5 molD	3969
TrwG/VirB8$_{peri}$ mol1	TrwG/VirB8$_{peri}$ mol2	419
TrwG/VirB8$_{peri}$ mol2	TrwG/VirB8$_{peri}$ mol3	464
TrwF/VirB9$_{NTD}$	TrwF/VirB9$_{NTD}$	781
TrwK/VirB4$_{central}$	TrwI/VirB6	94
TrwM/VirB3	TrwI/VirB6	330
TrwJ/VirB5	TrwI/VirB6 molA	1003
TrwJ/VirB5	TrwI/VirB6 molB	599
TrwF/VirB9$_{NTD}$ molB	TrwE/VirB10$_{NTD}$ α1	426
TrwF/VirB9$_{NTD}$ molE	TrwE/VirB10$_{NTD}$ α1	404
TrwE/VirB10$_{CTD}$ molA	TrwE/VirB10$_{CTD}$ molD	2269
TrwE/VirB10$_{CTD}$ molA	TrwF/VirB9$_{CTD}$ molB	1289
TrwF/VirB9$_{CTD}$ molB	TrwH/VirB7 molC	1030
TrwF/VirB9$_{CTD}$ molB	TrwE/VirB10$_{CTD}$ moln	717
TrwF/VirB9$_{CTD}$ molB	TrwF/VirB9$_{CTD}$ molE	484
TrwE/VirB10$_{CTD}$ molA	TrwE/VirB10$_{CTD}$ molk	349
TrwE/VirB10$_{CTD}$ molA	TrwE/VirB10$_{CTD}$ molh	255
TrwE/VirB10$_{CTD}$ molA	TrwE/VirB10$_{CTD}$ molF	231
TrwE/VirB10$_{CTD}$ molA	TrwF/VirB9$_{CTD}$ molo	148

SI Table 3. Strains, plasmids, constructs and oligonucleotides used in this study.

a. Strains used in this study

**Strain**	**Description**	**Reference**
E. coli Top10	F- mcrA Δ(mrr-hsdRMS-mcrBC) Φ80lacZΔM15 Δ lacX74 recA1 araD139 Δ(araleu)7697 galU galK rpsL (StrR) endA1 nupG. Used for cloning.	Invitrogen
E. coli BL21*(DE3)	F^- ompT hsdSB (rB^-, mB^-) galdcmrne131 (DE3) This strain is designed to overexpress high-levels of non-toxic proteins.	Invitrogen

b. Plasmids used in this study

**Plasmid**	**Description and Reference**	**Source**
pASK-IBA3C	Cloning vector provides chloramphenicol (Cam) resistance and carries a C-terminal Strep-tag. Protein expression is under the control of Tet promoter	Iba Life sciences
pCDFDuet-1 vector	A dual MCS cloning vector with spectinomycin (Spec) resistance and carries C-terminal His-tag and Strep-tag in MCS1 and MCS2 respectively. Protein expression is under the control of T7 promoter	Novagen
pET29b:hcp1$_{C\text{-}His}$	pET29b vector containing hcp1 gene with a C-terminal His6-tag. It provides kanamycin (Kan) resistance. Protein expression is under the control of T7 promoter.	Provided by Dr. Joseph D. Mougous
pMAK3	Wild-type T4SS conjugative plasmid of *Salmonella enterica*, share 97.3% DNA sequence identify with the pR388, which is also the wild-type T4SS conjugative plasmid	Lab material

c. Constructs used in this study

**Construct**	**Description and Reference**	**Source**	**FP RP**
IBA3C: trwMtrwK-L6-hcp1$_{C\text{-}Strep}$	C-terminal Strep-tagged TrwM (VirB3) and TrwK (VirB4) from *E. coli* R388 plasmid cloned in pASK-IBA3C plasmid, selected with chloramphenicol resistance and overexpressed under Tet promoter	This study	P1 P2<br>P3 P4
pCDFDuet-1: traB$_{C\text{-}His}$ traG$_{C\text{-}Strep}$	C-terminal His-tagged TraB (VirB4) and Strep-tagged TraG (VirB11) from *E . coli* pKM101 plasmid cloned in MCS1 and MCS2 respectively of pCDFDuet-1 plasmid, with spectinomycin resistance, overexpressed under T7 promoter.	This study	P5 P6<br>P7 P8<br>P9 P10<br>P11 P12
pCDFDuet-1: traB$_{C\text{-}His}$ traG (Q8D) $_{C\text{-}Strep}$	TraB (VirB4)-TraG (VirB11) interface mutant with mutation of Q8D on TraG.	This study	P13 P14

pCDFDuet-1: traB$_{C\text{-}His}$ traG (R54E) $_{C\text{-}Strep}$	TraB (VirB4)-TraG (VirB11) interface mutant with mutation of R54E on TraG.	This study	P15 P16
pCDFDuet-1: traB$_{C\text{-}His}$ traG (N55E) $_{C\text{-}Strep}$	TraB (VirB4)-TraG (VirB11) interface mutant with mutation of N55E on TraG.	This study	P17 P18
pCDFDuet-1: traB$_{C\text{-}His}$ traG (K58E) $_{C\text{-}Strep}$	TraB (VirB4)-TraG (VirB11) interface mutant with mutation of K58E on TraG.	This study	P19 P20
pCDFDuet-1: traB (E591R)$_{C\text{-}His}$ traG$_{C\text{-}Strep}$	TraB (VirB4)-TraG (VirB11) interface mutant with mutation of E591R on TraB.	This study	P21 P22
pCDFDuet-1: traB (E594R)$_{C\text{-}His}$ traG$_{C\text{-}Strep}$	TraB (VirB4)-TraG (VirB11) interface mutant with mutation of E594R on TraB.	This study	P23 P24
pCDFDuet-1: traB (A598E)$_{C\text{-}His}$ traG$_{C\text{-}Strep}$	TraB (VirB4)-TraG (VirB11) interface mutant with mutation of A589E on TraB.	This study	P25 P26
pCDFDuet-1: traB (Y619R)$_{C\text{-}His}$ traG$_{C\text{-}Strep}$	TraB (VirB4)-TraG (VirB11) interface mutant with mutation of Y619 on TraB.	This study	P27 P28
pBADM11_*trwN/virB1-trwE/virB10*-Strep_rbs_*trwD/virB11*_rbs_10xHis-*trwB/virD4*		Redzej et al 2017[1]	
pMAK3Δ*trwK/virB4*	pMAK3 plasmid with an ampicillin cassette inserted into *trwK/virB4* gene	This study	Rec1 Rec2
pMAK3Δ*trwI/virB6*	pMAK3 plasmid with a chloramphenicol cassette inserted into *trwI/virB6* gene	This study	Rec3 Rec4
pBADM11_*trwI/virB6*	TrwI (VirB6) from *E. coli* R388 plasmid cloned in pBADM11 plasmid, selected with carbenicilin resistance and overexpressed under ara promoter	This study	P29 P30 P31 P32
pBADM11_*trwI/virB6*$_{C\text{-}Flag}$	TrwI (VirB6) from *E. coli* R388 plasmid cloned in pBADM11 plasmid, selected with carbenicilin resistance and overexpressed under ara promoter	This study	P29 P33 P31 P32
pBADM11_*trwI/virB6*(T41F)$_{C\text{-}Flag}$	TrwI/VirB6 mutant with mutation of T41F	This study	P34 P35
pBADM11_*trwI/virB6*(G48I)$_{C\text{-}Flag}$	TrwI/VirB6 mutant with mutation of G48I	This study	P36 P37
pBADM11_*trwI/virB6*(V60E)$_{C\text{-}Flag}$	TrwI/VirB6 mutant with mutation of V60E	This study	P38 P39
pBADM11_*trwI/virB6*(Q118E-Q122E)$_{C\text{-}Flag}$	TrwI/VirB6 mutant with mutation of Q118E-Q122E	This study	P40 P41
pBADM11_*trwI/virB6*(Q118L-Q122F)$_{C\text{-}Flag}$	TrwI/VirB6 mutant with mutation of Q118L-Q122F	This study	P42 P43
pBADM11_*trwI/virB6*(N105D-Q108E)$_{C\text{-}Flag}$	TrwI/VirB6 mutant with mutation of N105D-Q108E	This study	P44 P45
pBADM11_*trwI/virB6*(N105L-Q108F)$_{C\text{-}Flag}$	TrwI/VirB6 mutant with mutation of N105L-Q108F	This study	P46 P47
pBADM11_*trwI/virB6*(Q222E-S226E)$_{C\text{-}Flag}$	TrwI/VirB6 mutant with mutation of Q222E-S226E	This study	P48 P49
pBADM11_*trwI/virB6*(Q222F-S226L)$_{C\text{-}Flag}$	TrwI/VirB6 mutant with mutation of Q222F-S226L	This study	P50 P51
pBADM11_*trwI/virB6*(T19W-T92W)$_{C\text{-}Flag}$	TrwI/VirB6 mutant with mutation of T19W-T92W	This study	P52 P53 P54 P55
pBADM11_*trwI/virB6*(T19W-A88W)$_{C\text{-}Flag}$	TrwI/VirB6 mutant with mutation of T19W-A88W	This study	P52 P53 P56 P57
pBADM11_*trwI/virB6*(D23W-A88W)$_{C\text{-}Flag}$	TrwI/VirB6 mutant with mutation of D23W-A88W	This study	P58 P59 P56 P57
pBADM11_*trwI/virB6*(Q15W-D96W)$_{C\text{-}Flag}$	TrwI/VirB6 mutant with mutation of Q15W-D96W	This study	P60 P61 P62 P63

d. Primers used in this study

**Primer name**	**Primer-Sequence (5'-3')**
P1	GGTAGCGGCTCTGGTAGCATGGCTGTTGATATGTTCATCAAG
P2	CCAAGCGCTCTCGAGGGCCTGCACGTTC
P3	CAGGCCCTCGAGAGCGCTTGGAGCCACCCGCA
P4	GCTACCAGAGCCGCTACCTACGTCGCTCCTTTCGGCTTTC
P5	TAACTGCAGGTCGACAAGCT
P6	GGTATATCTCCTTATTAAAGTTAAA
P7	ATAAGGAGATATACCATGAGAGCTGCCACCGCTAC
P8	GTCGACCTGCAGTTAGTGGTGATGATGGTGATGAGCC
P9	CATTCAGCATTTGCATGGTTTG
P10	AGGGCATCGTTCCCACTG
P11	TGGGAACGATGCCCTATGACTGATGCAGCTTTCTATCAAC
P12	TGCAAATGCTGAATGTTATTTTTCGAACTGCGGGTGGC
P13	TTTCTATGATCTTGGCCCACTGCGCGAGTATTTAGAAGATCCTACTGTTTTTGAAATTCGCATTAACTGCTTTCAGG
P14	CCAAGATCATAGAAAGCTGCATCAGTCATGATATCATAGATATCCAATTGAGATCTGCCATATGTATATCTCCTTCTTATACTTAACTAATATACTAAGATGGGGAATTGTTATCCGC
P15	TTTTATTGAAAACCTTGCTAAATCGTTGGTGAGC
P16	AGGTTTTCAATAAAATCTGCCGTAATTGCCGC
P17	TATTAGGGAACTTGCTAAATCGTTGGTGAGC
P18	GCAAGTTCCCTAATAAAATCTGCCGTAATTGCC
P19	CCTTGCTGAATCGTTGGTGAGCAGCAACAAG
P20	AACGATTCAGCAAGGTTCCTAATAAAATCTGCC
P21	GGCTACCCGTCTGGCTGAAGGCGTTGATGC
P22	GCCAGACGGGTAGCCATCGTTGCGCTG
P23	ACTGGCTCGTGGCGTTGATGCGGTTATGGG
P24	ACGCCACGAGCCAGTTCGGTAGCCATCG
P25	CGTTGATGAAGTTATGGGGGAAGGCTCACTTATTCCACGCG
P26	ATAACTTCATCAACGCCTTCAGCCAGTTCGGTAGCCATCG
P27	GGACGGGCGTGTGAATGAAGTTGTGGAAAATGGCGTATCACTGAAAGGGCTGCTGCGCGAATGGACGC
P28	TTCACACGCCCGTCCAGGATCGTAACGGTGCGCGCCTCGCGTGGAATAAGTGAGC
P29	CAGGAGGAATTAACCATGGCCTTCGAGCTGTTCACACCG
P30	AACAGCCAAGCTCTCTTACTACCCTGCTTTGCGTCCCCG
P31	GAGAGCTTGGCTGTTTTGGC
P32	GGTTAATTCCTCCTGTTAGCC
P33	AACAGCCAAGCTCTCTTACTAGAATTTATCGTCGTCATCCTTATAATCG
P34	GGGTTTGTTTCTTGGCTTCATTACCTATGGCTGG
P35	CCAAGAAACAAACCCACCGAAACAACCG
P36	CATTACCTATATTTGGCTCATTATTCGGGGCGCGGTGGAAATGC
P37	CGAATAATGAGCCAAATATAGGTAATGAAGCCAAGGGTCAAACCCAC
P38	AATGCCGGAAGCGGAATTTCTCAACCGCTGC
P39	TCCGCTTCCGGCATTTCCACCGCGCC
P40	ACGAATCGGCGGAACAAGGGTTTGACCGGGCAAGC
P41	GTTCCGCCGATTCGTCAACCAACGCGGCCGC
P42	ACCTGTCGGCGTTTCAAGGGTTTGACCGGGCAAGC
P43	GAAACGCCGACAGGTCAACCAACGCGGCCG
P44	CCTGCTCGGAGATCCGACAGAAGGGGCGTCGGCG
P45	GACGCCCCTTCTGTCGGATCTCCGAGCAGGGCCG
P46	GACTGCCGACATTTGGGGCGTCGGCGGCCGCG

P47	CAAATGTCGGCAGTCCGAGCAGGGCCGAAGC
P48	GCTGCTGATGGAAATATTCGGGGAATACATGGCCGATCTGCGGTTTGATG
P49	CGGCCATGTATTCCCCGAATATTTCCATCAGCAGCCCGAACACAGCCG
P50	GTTTATATTCGGGCTGTACATGGCCGATCTGCGGT
P51	AGCCCGAATATAAACATCAGCAGCCCGAACACA
P52	AACGGCATGGTATGTCACGGACATTTCCTCGCG
P53	ACATACCATGCCGTTGTCTGGTCAATCTTG
P54	TGCCATTTGGACGGTGCCGGATGAGCTG
P55	ACCGTCCAAATGGCATTAGCGATTTCGCCTTG
P56	CGAAATCTGGAATGCCATTACCACGGTGCC
P57	GCATTCCAGATTTCGCCTTGATACAAACCACCG
P58	TGTCACGTGGATTTCCTCGCGGGCAATCG
P59	GAAATCCACGTGACATACGTTGCCGTTGTC
P60	GATTGACTGGACAACGGCAACGTATGTCACG
P61	GTTGTCCAGTCAATCTTGTTGAAAAGCGG
P62	GGTGCCGTGGGAGCTGGCTTCGGCCCTG
P63	AGCTCCCACGGCACCGTGGTAATGGC
Rec1	GGAGACGCCGGTAGGCCAGTTCATTCCATACTCGCACCACGTTACTGACACGATCAATTAACCCTCACTAAAGGGCGGCCGC
Rec2	CCACACCGCCGGGTCGTCTCCGACTTCGGCAATAATGCTTTCGGCAATTTCGGCTAATACGACTCACTATAGGGCTCGAGGAAGTTCC
Rec3	CGCACCGCTGCGCATCGAGCGCAATTCCCACATATAGCCAAGGCCAATGCCATAATACGACTCACTATAGGGCTCGAG
Rec4	GTTCCAAGGCGACGGGATGCCAAAAATCCGGTAGGGAGGAAATTGCCGTGTAAAATTAACCCTCACTAAAGGGCGGCCG

Supplementary References

1 Redzej, A. *et al.* Structure of a VirD4 coupling protein bound to a VirB type IV secretion machinery. *EMBO J* **36**, 3080-3095, doi:10.15252/embj.201796629 (2017).
