# Supplementary material for: Cryo-EM structure of a type IV secretion system
Source: Nature. 2022 Jun 22;607(7917):191–6. doi: 10.1038/s41586-022-04859-y (PMC9259494; doi:10.1038/s41586-022-04859-y)
Supplement: Supplementary file 7 — Source Data Fig. 4 [file 41586_2022_4859_MOESM7_ESM.docx]

**Source Data**

Cryo-EM structure of a type IV secretion system

Kévin Macé, Abhinav K. Vadakkepat, Adam Redzej, Natalya Lukoyanova, Clasien Oomen, Nathalie Braun, Marta Ukleja, Fang Lu, Tiago R.D. Costa, Elena V. Orlova, David Baker, Qian Cong, and Gabriel Waksman

|  | Mating efficiencies relative to TrwI-Flag | | |
| --- | --- | --- | --- |
| Construct | First repeat | Second Repeat | Third Repeat |
| vector | 0 | 0.00022337 | 0 |
|  |  |  |  |
| Thr41Phe | 0.00257908 | 0.02651489 | 0.02602921 |
| Gly48Ile | 0 | 0.00013711 | 0.00030682 |
| Val60Glu | 0.00016224 | 0 | 0.00453707 |
|  |  |  |  |
| Gln118Glu Gln122Glu | 1.54187495 | 2.2688172 | 2.56844201 |
| Gln118Leu Gln122Phe | 4.25387841 | 3.64685004 | 3.42528527 |
| Asn105Asp Gln108Glu | 8.068906 | 0.9388419 | 3.59780481 |
| Asn105Leu Gln108Phe | 17.4340136 | 12.5469012 | 7.72635815 |
| Gln222Glu Ser226Glu | 1.68464782 | 0.76033058 | 1.53289253 |
| Gln222Phe Ser226Leu | 3.57957958 | 9.48778104 | 2.42630385 |
|  |  |  |  |
| Thr19Trp Thr92Trp | 0.15741303 | 1.35334286 | 0.67381221 |
| Thr19Trp Ala88Trp | 0.05908885 | 0.06003085 | 0.45661095 |
| Asp23Trp Ala88Trp | 0.00181776 | 0.0182205 | 0.0215223 |
| Gln15Trp Asp96Trp | 0.02728148 | 0.44128261 | 0.48920468 |

Source data for mating efficiencies of TrwI mutants relative to TrwI wild-type – Related to Fig. 4d
